# Social media narratives, diasporic identity and collective memory: A critical synthesis of the literature

**DOI:** 10.1177/14687968251386072

**Published:** 2025-10-03

**Authors:** Masoud Kianpour, Anna Triandafyllidou, Thomas Allen, Shiva Mazrouei, Morteza Shams

**Affiliations:** 1 Canada Excellence Research Chair program on Migration and Integration, 7984 Toronto Metropolitan University, Toronto, ON, Canada; 2 Department of English, University of Ottawa, Ottawa, ON, Canada; 3 3710 Department of Health, Aging & Society, McMaster University, Hamilton, ON, Canada; 4 Department of Communication, University of Tehran, Tehran, Iran

**Keywords:** social media narratives, diasporic identity, collective memory, digital storytelling

## Abstract

This study investigates the evolving relationship between social media narratives, diasporic identity, and collective memory in a context marked by increasing migration and growing digital media engagement. Employing a scoping review as a meta-analysis approach, we analyzed scholarly literature from 2014 to 2024 across Diaspora Studies, Sociology, and Communication Studies to understand how cultural and identity narratives are evolving amid fast-developing digital technologies. Out of 250 sources collected, 69 were shortlisted for in-depth review based on their relevance to the research questions. The study reveals a dichotomy in digital narratives concerning diasporic identity and collective memory, highlighting both positive potentials and negative drawbacks. On the positive side, digital narratives can foster empowerment, memory preservation, and community building. On the negative side, they may pose challenges to personal and collective identity, exhibit anti-democratic tendencies, and undermine cultural diversity. The research concludes by proposing a new analytical framework for examining diasporic identity and collective memory in relation to social media narratives, along with specific suggestions for future research in this ever-evolving ecosystem.

## Introduction

Contemporary society is shaped by an “advanced digital infrastructure”, characterized by interconnected devices, systems, and technologies that process, store, and transmit vast amounts of data (Government of Canada, 2024). Within this digital ecosystem, a new era of narrative production has emerged, driven first by mass media and later by social media, in which the large-scale creation and dissemination of information and narratives is not only possible but actively encouraged. The advent of Web 2.0 and social media has transformed how narratives are produced and shared by facilitating interactive engagement and dynamic digital storytelling. The rise of “prosumers” (Bruns, 2008)—individuals who produce and consume content at the same time—has led to a significant increase in the creation of digital content by a large number of people, including members of diasporic communities, who use social media to share their experiences, identities, and perspectives as they straddle different cultural, social, and geographical borders (Plascencia, 2018; Ponzanesi, 2020).

Echoing Brubaker (2005), we understand the term *diaspora* not as a fixed or bounded entity, but rather as an *idiom*, a *stance* or a *claim*. Diasporic communities are those that either self-identify or are characterized as maintaining a connection—real or imagined—to a homeland while living in a “host society.”^1^ These communities often navigate experiences of displacement, cultural negotiation, and varying degrees of marginalization, among both first-generation and subsequent generations. Members of diasporic communities frequently engage in transnational practices that link their homeland and host society—connections that are increasingly facilitated and diversified through advanced digital technologies. As Hage (2021) puts it, migrants used to experience home at a distance by imagination, but today they increasingly do so by communication.

Given the power of personal and community narratives on social media (Han, 2024), the mediatized world of the digital age significantly influences official and unofficial discourses, public opinion, and the construction of cultural norms and societal values (Barassi and Zamponi, 2020; Can et al., 2025; Lund et al., 2018). As Ponzanesi (2020:978) argues: “digital formations facilitate and transform the possibilities for diasporic affiliations.” Members of these communities increasingly leverage social media to facilitate key aspects of their immigration process, including integration and acculturation in host societies (Dayani, 2017; Lim and Pham, 2016; Mitra and Evansluong, 2019), identity reconstruction and negotiation (Glukhov, 2017; Sanyu, 2018), building social capital (Haug, 2008; Oh, 2016), and maintaining connections with their homelands while preserving cultural heritage (Bacigalupe et al., 2015; Collins, 2009).

However, much of the existing research on how new media and digital technologies shape diasporic identity and narratives remains fragmented within traditional disciplinary boundaries, leaving a gap in our understanding of this rapidly evolving field from an interdisciplinary perspective. Given that the functions and effects of social media are interrelated, a comprehensive review of the scattered research on the effects of digital narratives on diasporic identity and collective memory is both necessary and overdue. Additionally, existing studies tend to focus on diaspora-generated content, but less attention has been paid to outsider narratives about diasporic communities and their potential impact on identity formation. This research aims to bridge that gap by examining the evolving landscape of digital narratives and critically assessing how different studies interpret their consequences for groups identified as diasporic.

The central objective of this study is to examine how shared cultural and historical narratives are digitally constructed on social media, with a focus on diasporic communities and migrant populations. To achieve this, we conducted a scoping review of scholarly literature from the past decade, analyzing research on diasporic communities and their cultural aspects, while also considering other dimensions of identity, including religious, political, national, social, and citizenship identities. This research highlights the dual role of digital narratives in shaping diasporic identities— digital narratives afford opportunities for cultural expression and integration but also pose challenges such as identity fragmentation and digital amnesia. By synthesizing diverse perspectives from interdisciplinary fields, the study provides insights into the intersection of social media, digital storytelling, and diasporic identity formation. This study centers on a key research question: How do digital narratives on social media influence the cultural identity and collective memory of diasporic communities? To answer this, we conducted a meta-analysis of existing studies, taking it a step further by systematically reviewing and synthesizing their findings.

## Methodology

This project is based on a scoping review method, an exploratory study that surveys existing literature to identify key concepts, theories, and sources of evidence shaping the field (Romund, 2017). We employed a meta-analysis approach to examine theoretical and empirical trends concerning social media narratives, diasporic identity and collective memory. Our review focuses on scholarly literature from the past decade, identifying dominant theoretical and normative perspectives across several humanities disciplines with a particular emphasis on Communication and Media Studies and Diaspora Studies. To conduct this literature review, the following databases were utilized: Academic Search Complete (EBSCOhost), JSTOR, Oxford Journals Online, Sage Journals, ScienceDirect, SpringerLink, Scopus, Web of Science, PubMed, Sociological Abstracts, Worldwide Political Science Abstracts, Google Scholar, and Elsevier (Scopus).

The inclusion criteria focused on peer-reviewed scholarship published between 2014 and 2024. This review provides insights into how digital narratives on social media impact identity expression, community building, diasporic identity, and collective memory, highlighting both positive influences and negative drawbacks. We conducted searches using various keyword combinations, including: “social media narratives, digital storytelling, multiculturalism, collective memory, historical memory, technology, cultural narratives, identity narratives, advanced digital technology, transnationalism, globalization, diaspora, community mobilization, racialized minorities, cultural identity, belonging, integration, and citizenship.”

Overall, a total of 250 studies were initially collected in different formats, including peer-reviewed articles, book chapters, books, and policy briefs. Of these, 69 were shortlisted for in-depth analysis based on their relevance to our research questions. All selected documents were analyzed and summarized using qualitative data analysis. Texts were included if they substantively addressed at least one of the three core areas— social media narratives, diasporic identity and collective memory —and offered conceptual or empirical insight into identity construction in digital spaces. For inclusion in the final set and in-depth analysis, at least two of the three core areas had to be meaningfully engaged. We began with an initial thematic categorization, which was then refined inductively during the data analysis process. As part of this process, each text was summarized in a memo highlighting its main contributions and its relevance to the larger research questions.

To clarify further, this study employs a scoping review methodology, which differs from a systematic review in its purpose and approach. The purpose of a scoping review is to map the landscape of key literature related to a particular theme or research question, with an emphasis on identifying influential, conceptually rich, or field-shaping works, rather than achieving comprehensive coverage. Accordingly, the selection of texts was guided by their intellectual impact, relevance to the research focus, and, in some cases, their ability to catalyze broader public and scholarly conversations. While the review is not exhaustive—and some relevant and important works have been unintentionally omitted—the included texts were chosen because they have played a significant role in shaping discourse around social media narratives, diasporic identity, and collective memory. While we acknowledge that many other important studies remain to be explored, we have also included works that are not directly situated within Diaspora Studies (e.g., *Doppelganger* by Naomi Klein or *The Anxious Generation* by Jonathan Haidt). Such scholarship was included due to its thematic relevance and notable impact across both academic and public domains. These texts have sparked wide-ranging debates and have helped elevate scholarly concerns—particularly those related to identity, digital culture, and narrative—into broader cultural and societal conversations (Table 1).

**Table 1.** breakdown of reviewed sources.

Number of sources searched	Number of sources reviewed	Type of source			
		Peer-reviewed article	Book chapter	Book	Thesis and dissertation
250	69	48	6	11	4

## Digital technology and narratives

Narratologists have long understood that distinct layers of narrative activity operate in coordination to produce meaningful storytelling perceived by the listener or reader (Shklovsky, 2015). Identity formation in stories involves three key elements: narration, focalization, and agency (Bal, 2021). Narration refers to the storyteller, focalization determines the perspective through which the story is viewed, and agency identifies the character or actor who drives the action. In the context of digital storytelling on social media, Levine (2017) argues that networks facilitate connection and circulation, while narratives provide a framework for linking events over time. Social media enables the structuring of narrative roles—storyteller, focalizer, and actor—by organizing linguistic expressions such as status updates, tweets, and stories into temporal sequences. Furthermore, scholars have increasingly applied concepts from critical narratology and literary studies to analyze how individuals construct narratives of identity on social media. Unlike traditional media, social media platforms introduce distinct narrative structures that shape meaning through their unique affordances (Bucher and Helmond, 2018; Evans et al., 2016). For example, persistence ensures content remains accessible over time, while visibility determines who can see it. Spreadability allows narratives to circulate widely, and searchability helps users find relevant content. Interactivity fosters engagement through likes and comments, while anonymity and pseudonymity enable self-expression without revealing real identities. Overall, the formal constraints and interactive nature of social media platforms influence how individuals create, present, and negotiate their identities, highlighting the evolving relationship between storytelling, identity, and digital culture.

Storytelling on social media, often focused on daily life experiences, is selective, artistic, reflective, playful, and emotive (Mäkelä, 2019). While these narratives may lack historical reliability, their semi-fictional quality does not preclude them from shaping identity over time. We can thus talk about a parallel between literary and social media narratives, considering the fact that everyday posts coalesce into longer, enduring narratives of individual and collective identity. Such narratives rely on literary techniques such as analepsis, prolepsis, and anachrony. Georgakopoulou (2017) highlights the fragmented nature of digital storytelling, where readers must recall previous “small stories” (e.g., status updates) and construct narrative order from dispersed content. This challenges Han’s (2024) assertion that social media posts are not “genuine stories” due to their lack of “narrative duration.” The divergence in perspectives suggests a need for further research on whether social media narratives can achieve the depth and temporal structure of literary narratives.

Initially, scholarship emphasized the positive role of new media in producing and sharing narratives. The rise of mass and broadcast media, combined with Web 1.0, enabled narratives that contested dominant power structures, leading to a “war of narratives” (Ninan and Sergeeva, 2022). During the late 20^th^ and early 21^st^ centuries, narratives became tools of influence and propaganda, aligning with postmodern theories such as Lyotard’s (1979) concept of the decline of grand narratives. Instead of overarching meta-narratives, individuals turned to localized, fragmented narratives reflecting diverse perspectives.

With the advent of Web 2.0 and interactive features, individuals gained greater agency in producing and engaging with digital narratives. Castells (2009) introduces the concept of “mass self-communication,” which describes self-generated messages shared with self-selected audiences. This framework underscores the shift toward personalized digital storytelling. Koenitz’s (2010) Interactive Digital Narrative Theory further highlights how interactivity—absent in traditional mass media—shapes modern storytelling on digital platforms.

For diasporic communities, digital technology facilitates transnational connectivity. Parham (2004) argues that the Internet reduces temporal and spatial constraints, allowing diasporic groups to create shared virtual spaces. Keles (2016), drawing on Pantham’s social capital theory, emphasizes that information flow within social networks enhances social capital, enabling diasporas to strengthen their real and virtual communities. Inspired by Holgate et al.’s (2012) concept of “diasporic consciousness,” Kelz suggests that digital narratives help migrants consolidate resources and foster human, social, and economic capital.

A utopian perspective on digital communication posits that social media fosters democracy, cultural preservation, and identity reinforcement among marginalized groups (Batool et al., 2021; Jha and Kodila-Tedika, 2020; Wijaya et al., 2018). However, emerging research highlights concerns regarding digital technology’s impact on collective identity and memory. Geertz (1973) defines culture as “webs of significance or systems of meaning embodied in symbols.” The digital age accelerates the fragmentation and reconfiguration of these symbolic webs, leading to a lack of stable consensus on values, norms, and collective narratives. In this evolving landscape, multiple narratives and cultures coexist, and competing “present pasts” (Huyssen, 2003) vie for influence, often generating insecurity and moral outrage.

New theoretical frameworks, such as post-truth theory within critical communication studies, address such contemporary digital challenges. For example, Harsin (2018) introduces the concept of “attentional capitalism,” as a contemporary mode of governance in which perception is manipulated through hoax-ridden journalism, sophisticated data analytics, deceptive AI, microtargeted messaging, and promotional exaggeration. This environment fosters skepticism toward truth claims, leading to the rise of “emotional truth” or “emo-truth”—narratives that resonate as authentic despite their potential distortions. Likewise, Dixon (2016) introduces “the Social Media Stereotyping Model” (SMSM), arguing that while social media perpetuates racial stereotypes and social divisions, these processes are mediated by users’ specific social media consumption habits. His findings highlight the need for critical engagement with digital narratives to mitigate biases and promote a more inclusive representation of identity.

In such a data-driven landscape, further research is needed to understand how social media shapes the identity and collective memory of diasporic communities. Exploring this dynamic will shed light on digital narratives as both empowering tools and contested spaces in contemporary society. The influence of digital power and connectivity on diaspora identity extends beyond the traditional homeland-host society divide. By emphasizing the role of “place,” social media enables diaspora members to engage across geographic boundaries, actively shaping their collective identity and memory.

## Diasporic identity and collective memory

The intersection of identity and narrative is encapsulated in the term “narrative identity,” which refers to how individuals construct and understand their sense of self through the stories they tell about their lives (McAdams and McLean, 2013; Ricoeur, 1991). Narrative identity functions as a form of autobiographical memory and a psychological resource for self-understanding, social connection, and future direction. In diaspora studies, identity and its various aspects have always been of central concern (Georgiou, 2010; Tsolidis, 2014). Ponzanesi (2020) suggests that “diaspora refers to a post-national space that complicates the relationship between nation, land, and identity.”

Digital technologies have profoundly impacted contemporary societies, offering an unprecedented array of opportunities for diasporic communities to explore and articulate their identities as they provide avenues for discussion and debate on issues of belonging and identity (Hirji, 2006). Research indicates that social media plays a crucial role in shaping immigrant experiences and perspectives (Plascencia, 2018) and that digital networks impact the identity orientations of immigrants (Aghapouri, 2020).

Marino (2015), in her study on Italian immigrants in England, found that social media function as integrated virtual spaces where immigrant communities form and negotiate transnational identities, fostering a pattern of creative media consumption. Digital technologies empower immigrants to participate in public affairs, improve their living conditions in the host country, and engage in various social and political activities (Bernal, 2020). Studies also show that diaspora communities utilize social media to maintain and strengthen their ethnic and national identities, establish connections with their homeland, resist assimilation, and simultaneously facilitate integration into their host country’s identity (e.g., Croucher and Rahmani, 2015; Lim, 2022). By providing digital connectivity and content-sharing opportunities, social media has transformed the activism of diaspora communities regarding their identity and narratives, influencing the formation of their collective memory. Digital platforms now mobilize memory to preserve and commemorate events, creating what is known as “digital power” (Ossé and Massicotte, 2021) or “cybertouch” (Kuntsman, 2010).

## Social media: A double-edged sword

While social media has been praised for bridging geographical and cultural gaps, enhancing access to resources and knowledge and helping diaspora members navigate different socio-political environments, it has also faced criticism. Critics argue that it fosters superficial connections, contributes to information overload, and undermines meaningful relationships and collective memory formation. The early vision of a digitally facilitated global village is now obscured by concerns over the increasing influence of algorithms, artificial intelligence, and digital surveillance on identity formation (Klein, 2023; Lupton, 2016).

**Figure 1.**
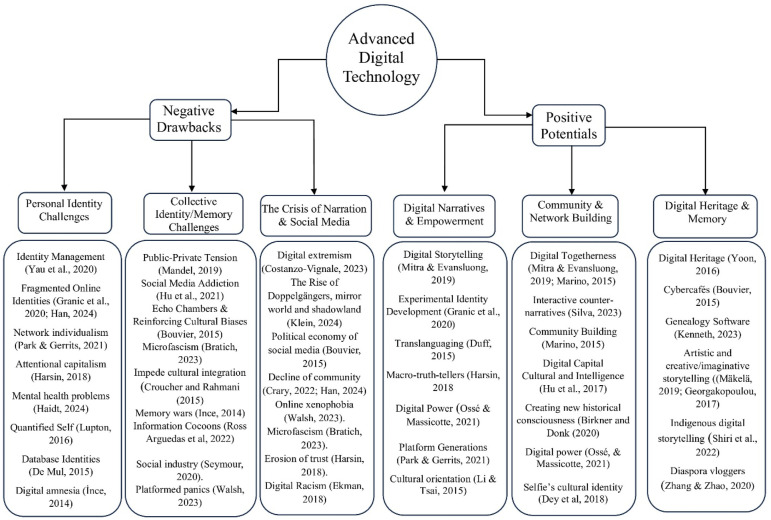
The impact of digital narratives on diasporic identity and collective memory.

While earlier studies highlighted the empowering potential of social media through concepts such as “second life” (Turkle, 2005), “virtual communities” (Rheingold, 2000), and “online activism” (Tufekci, 2017), more recent research critiques these platforms as spaces that foster misinformation, manipulation, and digital alienation. Scholars now describe social media environments as dominated by “doppelgangers” (Klein, 2023), “micro-fascists” (Bratich, 2023), and such digital maladies as fake news, rumor bombs, microtargeting, neuromarketing, bots, and trolls (Harsin, 2018). Once considered platforms for fostering narrative identity, spontaneous storytelling, and counter-narratives (Granic et al., 2020) these online spaces are now increasingly described as the “nude internet” (Coaston, 2024), where trolling, performative engagement, and digital narcissism thrive (Seymour, 2020). This evolving dynamic invites further exploration of its impact on both personal and collective identity formation (Figure 1).

## Positive potentials

### Digital narratives & empowerment

Through digital narratives and storytelling, migrants construct a dynamic narrative identity that bridges personal memories with future aspirations. This process is fluid, shaped by interactions in digital spaces where individuals selectively present aspects of their identity based on audience expectations and platform affordances (Parks and Gerrits, 2021). Such digital engagement enables migrants to navigate their transitions in the host country, maintain connections with their heritage culture, and adapt to host society norms. For instance, active participation on social media fosters communication with both home and host communities, reinforcing a sense of belonging and facilitating integration (Mitra and Evansluong, 2019).

Social media platforms have become significant arenas for identity experimentation, particularly among adolescents and young adults. These digital environments allow individuals to explore different roles and personas, a critical process in identity development (Granic et al., 2020). They provide liminal spaces (Mitra and Evansluong, 2019), enabling users to express multiple facets of their identity in ways that transcend real-world constraints. Mitra and Evansluong (2019) argue that social media offers immigrants a safe space to navigate and reconcile their cultural identities while sustaining connections to their heritage. Similarly, Dey et al. (2018) explore how young British South Asian adults use social media to articulate and manage their dual cultural identities. Their research illustrates how digital interactions shape self-expression in multicultural settings, often through visual symbols. For example, diaspora members use selfies with cultural icons—such as cricket and Bollywood stars—to construct and reinforce their cultural identities, engaging in digital exchanges with peers and family to validate these identities.

Social media platforms offer various affordances (Bucher and Helmond, 2018; Evans et al., 2016) that empower users to shape and redefine their identities. The ability to share personal stories and reach vast audiences allows migrants to create and disseminate digital narratives, enhancing their agency and visibility. This “digital power” (Ossé and Massicotte, 2021) provides individuals with the means to highlight their experiences, craft self-representations, and engage with different communities. The flexibility of digital spaces enables users to edit, rename, and modify aspects of their identity—ranging from visual aesthetics to ideological affiliations—ultimately shaping narratives of who they were, who they are, and who they aspire to become.

Members of diaspora strategically leverage different platforms to express layered aspects of their identity, tailoring their self-representation based on audience expectations and technological affordances. This selective self-presentation illustrates how individuals negotiate influences from both host and home cultures while managing the perceptions surrounding their digital narratives (Parks and Gerrits, 2021). Social media provides the flexibility to engage in both public and private identity performances, granting users control over how much of their personal and cultural identity they reveal to different audiences (Mandel, 2019).

Beyond personal identity formation, social media interactions contribute to broader cultural shifts. These platforms enable users to construct, maintain, or reimagine cultural communities in ways that challenge traditional institutions (Bouvier, 2015). As digital spaces become central to social relations and identity management, they facilitate new forms of cultural negotiation (Shiri et al., 2022; Zhang and Zhao, 2020), allowing individuals to take control of their narratives while reshaping social and political discourse.

An emerging body of literature examines how social media shapes narratives about migrants and refugees, particularly in immigrant-receiving countries like Canada (Triandafyllidou and Monteiro, 2024). In this regard, a prominent example is Canada’s “immigrant exceptionalism” narrative, which portrays the country as a welcoming and inclusive space for newcomers. This narrative is reinforced through social media and is characterized by: (1) A high level of regular immigrant admissions through a structured immigration system; (2) Broad political and public consensus on the necessity of immigration; (3) Multiculturalism policies promoting the inclusion of diverse newcomers; (4) A human rights-based legal framework for refugee protection (Monteiro, 2024). These narratives shape how newcomers interpret their experiences, influencing their identity construction and social positioning.

### Digital heritage and memory

The memories shared and passed down within diasporic communities play a crucial role in shaping one’s sense of self and belonging. The transmission of stories and experiences across generations fosters a shared historical consciousness, influencing how immigrants understand their past and present identities. Scholars highlight the positive role of social media in this process, arguing that digital platforms enable both individual and communal engagement with cultural heritage in unprecedented ways (Marino, 2015). Unlike traditional communication methods, which often hindered the transformation of personal immigrant narratives into collective memory over time, today’s social media allow personal stories to gain viral traction, overcome temporal and spatial barriers, and rapidly evolve into widely shared narratives. Consequently, the boundary between private and public memory is becoming increasingly blurred (Kozdras et al., 2015).

Social media, as a tool for social organization, enables diasporic communities to reconnect, expand their networks, and foster what Kozdras et al. (2015) describe as “communities-in-the-making.” The ability to increase the size and diversity of these networks and to widely disseminate cultural narratives has been conceptualized as a form of “digital capital” (Lusis, 2012). This digital capital enhances the capacity of diaspora members to sustain their heritage while simultaneously integrating into new societies. Research underscores the role of digital technology and new media in preserving collective memory. Through the creation of a “digital heritage” (Yoon, 2016), immigrants utilize digital platforms to document and share cultural artifacts, traditions, and historical narratives. Activities such as digital archiving, online exhibitions, and interactive platforms broaden access to cultural materials, reaching diverse audiences. These digitalized memories create immersive experiences that evoke strong emotional connections and deepen diaspora members’ engagement with their shared histories.

Birkner and Donk (2020) argue that social media facilitates the emergence of “subordinate spaces of discourse” around historical memory, allowing alternative- and counter-narratives to challenge mainstream interpretations. These platforms empower immigrants to actively participate in historical discourse, shaping memory construction beyond state-sanctioned narratives. By providing immediate and interactive engagement with the past, social media fosters a “new historical consciousness,” incorporating diverse voices into collective memory. Studying digital discourses offers insights into the contested nature of collective memory and the evolving historical consciousness of civil society.

Social media plays a pivotal role in enabling migrant communities to maintain connections with their heritage while fostering integration into host societies. The concept of “digital togetherness” (Mitra and Evansluong, 2019) describes how migrants use online platforms to sustain cultural ties while cultivating new social relationships. These digital spaces facilitate identity negotiation, allowing individuals to balance their heritage and host cultures (Marino, 2015). By sharing experiences, providing mutual support, and sustaining cultural practices, social media strengthens migrants’ sense of belonging and community.

Park and Gerrits (2021) explore how different generations of online social networks (OSNs) shape collective identity formation among Korean migrants in Germany. They introduce the concept of “platform generation,” categorizing OSNs into three phases: Web 1.0 (Private/Closed) – Focuses on heritage preservation and close-knit communities, where relationships extend beyond the digital space but remain limited to specific affiliations, such as ethnic identity within a national context (e.g., Korean migrants in Germany); Web 2.0 (Semi-Private/Semi-Closed) – Encourages dynamic community engagement, fostering increased interaction and participation among diaspora members; Web 3.0 (Public/Open) – Promotes a more flexible understanding of identity, allowing individuals to cultivate broader connections that transcend specific cultural or national affiliations.

### Community & network building

Social media serves as a crucial tool for migrants to engage in reciprocal communication with both co-nationals and locals, developing connections across multiple cultural contexts. Some scholars argue that such engagement strengthens identification with host culture values, supporting a more integrated social identity (Mitra and Evansluong, 2019; Silva, 2023). By participating in digital spaces, migrants can build relational networks that reinforce their sense of belonging. When these online interactions are met with positive reception, they enhance social integration; conversely, negative responses can adversely impact offline belonging, illustrating the interconnectedness of digital and real-world experiences (Mitra and Evansluong, 2019).

In *Mediating Migration*, Hegde (2015) explores how migrants’ experiences are shaped by media, communication technologies, and the broader socio-political conditions of globalization. This book argues that everyday media practices are deeply entangled with transnational migration: migrants do not simply consume media, but through remittances, social media, activism, political debate, and other mediated networks they reshape their identities, maintain ties across distances, challenge discourses of legitimacy, and reimagine what belonging means. Hegde shows how migrants and diasporic communities “connect” nations and negotiate what is considered authentic or worthy of recognition.

Social media provides migrants with tools to control access to cultural content, shaping how they remember their heritage and represent their identities. Through online content management, individuals selectively present aspects of their cultural identity, tailoring engagement to diverse audiences in ways that reinforce cultural belonging and cohesion. Yau et al. (2020) identify four key strategies migrants use to navigate identity on social media: (1) Regulating the Separation Between Home and Host Cultures – Controlling how much of each cultural identity is expressed in different contexts; (2) Managing Audience Access – Restricting who can see or engage with particular content; (3) Curating Shared Content – Selecting which cultural elements to emphasize or conceal in online spaces; (4) Integrating Offline and Online Experiences – Using digital platforms to reinforce lived cultural experiences.

These strategies highlight the reciprocal relationship between social media and identity, where digital platforms not only serve as spaces for expression but also actively shape the process of identity negotiation. For instance, the concept of “digital togetherness” is applied to Somali refugees’ use of social media in cybercafés, allowing them to sustain connections with established communities while navigating new environments (Bouvier, 2015).

Li and Tsai (2015) argue that social media platforms challenge traditional notions of cultural belonging. For instance, while Facebook originated in the U.S., it is no longer strictly an American platform; instead, it fosters diverse cultural value systems, making it a significant factor in acculturation models. These scholars suggest that the traditional binary classification of media into “mainstream” and “ethnic” categories is no longer applicable, as social media has blurred these distinctions. This shift necessitates a reconsideration of how digital media influences the acculturation process, particularly for migrants navigating multiple cultural affiliations.

The relationship between social media use and cultural integration is complex. Croucher and Rahmani (2015) examined Muslim immigrants in the U.S. and found that those who primarily used Facebook for interactions within their ethnic in-group were less motivated to adapt to the dominant U.S. culture. Moreover, increased Facebook usage among these individuals was correlated with more negative perceptions of the host culture. These findings suggest that while social media fosters community support and homeland connections, it may also reinforce ethnic identity at the expense of integration. This dual function underscores the nuanced role of platforms like Facebook in shaping immigrant experiences, both as enablers of cultural retention and potential barriers to full cultural integration.

Traditional migration studies have often conceptualized migration as a binary phenomenon—moving from an origin to a settlement. However, modern transnationalism presents a more complex picture, involving multiple dwelling points and sustained virtual and psychological connections beyond physical mobility (Duff, 2015). The emphasis has shifted from first-generation adult migrants to the experiences of children and youth, whose mobility is not only geographical but also digital. Social media platforms and chat rooms provide spaces for the exploration and representation of transnational identities, facilitating connections across borders and fostering hybrid, multilingual identities.

One notable aspect of this process is translanguaging, the blending of languages in everyday communication and artistic expression, which is especially prevalent among migrant youth (Duff, 2015). Social media provides a space where linguistic hybridity can thrive, reinforcing both cultural retention and the development of new, fluid identity formations. These digital interactions help young migrants balance their heritage language with the linguistic norms of their host culture, further complicating traditional notions of linguistic assimilation.

In a similar vein, Jørgensen and colleagues (2015) offer an empirical perspective on how migrants and young people in superdiverse societies navigate and negotiate identity and memory through their language practices, including in online spaces. Through the concept of *polylanguaging*, they show that speakers do not simply switch between bounded “languages,” but instead draw flexibly on linguistic *features* that carry shifting social and cultural values. This approach highlights how individuals co-construct belonging and self-presentation across interactions, whether face-to-face or digital, where multilingual repertoires are especially visible and fluid. These researchers stress that analyzing at the level of features reveals how language is both dynamic and negotiable, capturing processes of positioning and identity work that would be obscured by the traditional notion of separate “languages.” At the same time, they underscore the continuing political and institutional weight of “national languages,” which shape education, citizenship policies, and everyday categorization of migrants. For online contexts, as we saw above, this insight is particularly relevant: digital platforms allow migrants to blend features across linguistic repertoires to express identity, share memory, and negotiate inclusion or exclusion in transnational communities, even as broader social systems still impose rigid language boundaries. This study thus demonstrates how migrants’ digital practices of polylanguaging serve as powerful resources for co-constructing identity and memory in superdiverse environments.

Beyond identity construction, social media plays a crucial role as a source of information and social support for navigating new societies. This is particularly evident for refugees and immigrants, who use digital platforms to build networks and access essential resources that facilitate their adjustment process (Baumert et al., 2024). For example, among Syrian refugee youth in Canada, social media serves as a vital tool for resettlement, providing emotional support, information, and a sense of autonomy in their new environment (Ahmed et al., 2020). Additionally, social media can complement existing settlement services, alleviating some of the burden on formal institutions by enabling self-guided integration.

Dovchin and Izadi (2023) situate social media as a crucial arena for examining how migrants and communities in the Global South navigate identity, belonging, and memory through their everyday language practices. Rejecting fixed categories such as “bilingualism” or “multilingualism,” they highlight the *languaging turn* in discourse studies, which attends to fluid, semiotically rich practices that cut across languages, scripts, and modes. They argue that what is often treated in the Global North as “exceptional” or “creative” translingual practices are, in fact, ordinary, mundane, and normative for many Global South communities.

Increasingly, different scholars call for a decolonial perspective that challenges Eurocentric framings of linguistic diversity and foregrounds the everyday realities of Global South netizens. For example, Nguyễn and colleagues (2022) call for research on diasporic communities that moves beyond Anglocentric assumptions. They argue that English-centered perspectives obscure the linguistic and cultural diversity of diasporic lives, and instead highlight how identity and belonging are negotiated through multilingual and multimodal languaging. By situating diasporic practices within transnational and decolonial contexts, they show how migrants mobilize diverse repertoires to sustain ties, co-construct community, and resist linguistic hierarchies. In fact, by looking at diverse contexts, we can see how migrants and marginalized groups use digital platforms not only to express identity but also to negotiate power, resist exclusion, and engage in political and cultural participation.

Ultimately, languaging on social media can be seen as a site where the “sameness of difference” (Dovchin and Izadi, 2023: 1) emerges: practices that are at once deeply local and transnational, personal and collective. For migration and digital studies, this perspective underscores how online languaging becomes a resource for co-constructing identity and memory while simultaneously confronting global inequities.

## Negative drawbacks

While digital narratives on social media offer significant benefits for diasporic communities, our research also highlights their negative and potentially harmful impacts. These challenges can be categorized into three main themes: Personal Identity Challenges, Collective Identity/Memory Challenges, and Narrative Challenges. The following section explores these aspects in detail, examining how digital narratives may complicate identity formation, disrupt social cohesion, and contribute to hostility toward diasporic communities.

### Personal identity challenges

Digital interactions present significant challenges for authentic identity expression (De Mul, 2015; Hu et al., 2017, 2021). The performative nature of social media often pressures migrants to conform to perceived norms from both their home and host cultures, complicating identity management (Yau et al., 2020). Scholars argue that the public nature of social media further disrupts personal narratives, as users must navigate between private and public spheres of self-representation (Mandel, 2019). Additionally, the constant influx of digital content can diminish meaningful connections, leading to fragmented online identities (Granic et al., 2020). While social media facilitates storytelling and cultural exchange, it simultaneously forces migrants to negotiate identity within a globalized, hyper-connected environment where traditional identity frameworks may no longer apply (Belay, 2018).

The structure of online social networks (OSNs) further complicates identity formation. Unlike offline relationships, which often involve strong, enduring connections, OSNs are built on weak, temporary ties and are characterized by “network individualism” (Park and Gerrits, 2021). These networks prioritize instant interactions based on shared interests rather than long-term affiliations, reinforcing a fragmented sense of self. Additionally, the competitive nature of digital platforms—each vying for users’ attention—pressures individuals to navigate multiple online spaces with varying social norms, expectations, and values. As a result, social media fosters fragmented identities among diaspora members, creating barriers to integration and social cohesion. The emphasis on engagement and digital presence often overrides self-reflection, hindering the formation of a cohesive narrative identity, which is essential for a stable sense of self (Han, 2024).

One of the most prominent critiques of social media’s impact on personal identity comes from Naomi Klein’s (2023) *Doppelganger: A Trip Into the Mirror World*, in which she explores the distorted reality created by digital media. Klein introduces concepts like “mirror world” and “shadowland” to describe an environment where algorithmic curation, AI-generated content, deepfakes, and autocorrect features shape public perception. She argues that this “mirror world” not only reflects but also amplifies societal anxieties, producing a “doppelgänger effect”—where online personas and narratives often diverge sharply from real-life experiences.

Seymour (2020) critiques the role of social media in shaping public discourse and individual behavior, arguing that platforms like X amplify social polarization, outrage, and superficial engagement. His concept of the “social industry” describes how X’s design encourages reactionary, impulsive interactions at the expense of deep, critical thought. He further explores the implications of this “twittering” culture on democracy, mental health, and social relationships, suggesting that these platforms foster addictive behaviors while fragmenting public discourse. Similarly, Han (2017) describes how social media transforms collective behavior into a decentralized, impulsive swarm-like dynamic. Unlike traditional communities bound by shared goals or values, swarm behavior lacks long-term solidarity and is highly susceptible to manipulation. Han argues that this structure erodes individual autonomy, amplifies emotional volatility, and intensifies societal fragmentation. Within diasporic communities, these decentralized social media dynamics can weaken collective solidarity and reinforce individualism over communal identity, making it harder for migrants to cultivate a unified sense of belonging.

The performative nature of social media further complicates self-expression, as individuals curate and manage their online identities based on audience feedback (Mandel, 2019). This performative identity management creates a tension between authenticity and social conformity, where users feel pressured to align with dominant cultural expectations, trends, or social validation. Han (2024) describes this phenomenon as the “crisis of narration”, where narratives become overloaded with fragmented voices and disconnected information, ultimately lacking coherence and depth. This crisis not only affects individuals’ ability to construct meaningful self-narratives but also contributes to stress and anxiety, particularly among young people who must navigate multiple cultural influences (Granic et al., 2020; Haidt, 2024). For diasporic communities, the pressure to manage multiple identities—balancing their homeland heritage with host society expectations—can lead to identity confusion, instability, and emotional distress.

### Collective identity/memory challenges

Unlike traditional media, which provides a stable and centralized form of memory, new digital media encourages a fragmented and participatory approach to memory-making (İnce, 2014). This shift has enabled individuals to document and share events in real time, a phenomenon known as “digital witnessing,” which contributes to the formation of collective memory. However, the rapid dissemination and ephemeral nature of digital content challenge the sustainability of memory, often leading to fragmented and short-lived narratives among diasporic communities.

The fragility of digital storage, coupled with swift technological advancements and the obsolescence of storage formats, raises concerns about a potential “Digital Dark Age,” where essential cultural memories may be lost or distorted. İnce (2014) highlights the risks of manipulation and misinformation in digital spaces, which can significantly impact diasporic collective memory. Additionally, the ease of altering or fabricating digital content poses a serious challenge to the authenticity and reliability of cultural narratives. As digital memory shifts from centralized to decentralized structures, a plurality of voices emerges. While this inclusivity can enrich cultural heritage, it also generates conflicting narratives and contested histories, leading to “memory wars” (İnce, 2014), where digital platforms become battlegrounds for competing perspectives, often shaped by political agendas.

Social media algorithms further complicate collective memory formation by reinforcing polarized narratives (Leurs et al., 2020). While digital platforms serve as essential spaces for public discourse on immigration (Brekke and Thorbjørnsrud, 2020), their algorithmic designs prioritize engagement over balanced representation. By amplifying specific viewpoints while marginalizing others, these platforms contribute to a skewed and polarized perception of migration and diaspora experiences (Bouvier, 2015).

A particularly concerning factor is the role of AI-driven recommender systems, which act as digital gatekeepers (Bozdag, 2013). These algorithms are designed to increase user engagement by selectively filtering content, shaping users’ exposure to information in ways both selective (Alatawi et al., 2021; Dylko, 2016) and incidental (Heiss and Matthes, 2019; Weeks et al., 2017). By recommending content aligned with pre-existing interests while filtering out opposing viewpoints, these systems restrict information diversity (Berman and Katona, 2020), fostering echo chambers and filter bubbles (Pariser, 2011; Sunstein, 2017). Research suggests that such algorithmic curation limits exposure to alternative perspectives, intensifying polarization within immigrant communities and eroding trust between diasporic populations and host societies (Eg et al., 2023). This ultimately weakens the potential for shared collective identity formation, posing barriers to diaspora integration.

Furthermore, AI and machine learning algorithms carry implicit biases that shape public perception and behavior (Dolata et al., 2022; Mehrabi et al., 2022). Concerns surrounding fairness, accountability, and democratic integrity are central to discussions on the broader socio-political implications of algorithmic filtering (Persily and Tucker, 2020; Terren et al., 2021). Theories such as Echo Chambers (Sunstein, 2017), Information Cocoons (Ross Arguedas et al., 2022), and Filter Bubbles (Pariser, 2011) portray a dystopian future for social media’s algorithmic influence (Huszár et al., 2022; Pappalardo et al., 2024).

The political economy of social media plays a crucial role in shaping how diasporic identities and collective memories are constructed. From a media economics perspective, social media platforms are profit-driven entities, prioritizing user engagement and data commodification over authentic social interaction (Jin, 2018). The culture of connectivity fostered by these platforms primarily serves commercial interests, often promoting sensationalism, divisive content, and misinformation to maximize traffic and advertising revenue. These dynamics can skew public discourse on migration, reinforcing stereotypes, biases, and xenophobic narratives, rather than encouraging genuine cross-cultural understanding (Bouvier, 2015).

In this environment, social media not only reshapes public attitudes toward immigrants but also reconfigures how diasporic groups perceive themselves. Instead of fostering inclusive dialogue, these platforms frequently entrench ideological silos, reinforcing nationalistic sentiments or cultural biases. This further hinders integration efforts, as diaspora members struggle to mediate between their heritage and host societies in a digital landscape dominated by profit-driven algorithms.

While digital platforms enable community engagement through hashtags, online activism, and virtual storytelling, they also lack standardized methods for long-term preservation. Unless users take proactive steps to archive cultural content, critical aspects of diasporic heritage risk being lost in the constantly shifting digital landscape. Scholars such as Belay (2018) argue that globalization is not creating a “global village” but rather a “global metropolis”—an interconnected yet fragmented world where dominant digital narratives overshadow local identities and traditions. This dynamic aligns with the concept of “electronic colonialism,” where digital technologies facilitate rapid information exchange but also cultural homogenization. In this context, Crary (2022), in *Scorched Earth: Beyond the Digital Age to a Post-Capitalist World*, critiques the illusion of social media as a vehicle for real change. He argues that corporate-controlled networks cannot coexist with a sustainable, equitable society, emphasizing the incompatibility between digital capitalism and authentic human connection.

### Social media and the crisis of narration

Han (2024), critiques digital technologies for creating a “crisis of narration.” According to Han, modern readers no longer engage deeply with narratives. The “long, slow, lingering gaze” that once fostered introspection and daydreaming has been replaced by constant digital overstimulation. This shift results in what he terms “disengaged engagement”—a paradox in which individuals appear connected but consume narratives superficially, reducing them to mere information and commodities. Han warns that this crisis leads to a fragmented, incoherent narrative culture, where a flood of disjointed stories overwhelms individuals, preventing them from forming meaningful, sustained connections with identity and memory.

Amid this crisis of narration, social media platforms have introduced new challenges for diasporic communities, particularly in the spread of anti-immigration discourse. Research indicates that social media fosters xenophobia and intergroup antagonism, threatening multiculturalism by providing a space where anti-immigrant narratives proliferate (Walsh, 2023). These platforms have become battlegrounds for conflicting narratives, with immigration supporters and opponents clashing online (Triandafyllidou and Monteiro, 2024).

One critical aspect of this narrative warfare is the impact on constitutive narratives—the digital expressions of cultural and ethnic identity that promote tolerance, inclusivity, and historical preservation for diasporic communities. However, these narratives often face opposition from anti-immigrant discourse, which functions as a disruptive force and is weaponized in political campaigns to incite hate speech and xenophobia (Pitropakis et al., 2020). The rise of digital activism in response to these narratives has led Ekman (2018) to coin the term “Digital Racism”—a phenomenon in which online platforms serve as amplifiers for racial hostility and exclusionary ideologies.

Bratich (2023), in *On Microfascism: Gender, War, and Death*, emphasizes the central role of social media in fostering extremist ideologies. He argues that the digital landscape has become a recruitment ground for far-right radicalization, with online spaces mobilizing individuals to defend White supremacist ideologies (Costanzo-Vignale, 2023; Gagnon, 2020). In this context, anti-immigrant discourse is often structured around “platformed panics”—a term introduced by Walsh (2023) to describe how social media’s technical affordances, design, and algorithmic biases create an environment where moral panics against immigrants thrive.

Social media’s structural design further reinforces these dynamics by enabling far-right groups to cultivate collective identities around shared grievances. Anti-immigrant rhetoric is strategically amplified through digital echo chambers, where like-minded individuals reinforce exclusionary narratives. As a result, the politicization and polarization of immigration debates escalate, fueling hostility both online and offline (Ahmed et al., 2021; Bozdag, 2013; De Saint Laurent et al., 2020).

## Conclusion

In this scoping review and meta-analysis we tried to come up with new analytical reflections on the relationship between digital narratives and diasporic communities. We argued how the compression of the temporal and the spatial in identity narratives at a time of pervasive social media requires new conceptual frameworks to understand migrant identity and collective memory. We argued stories and narratives in these digital platforms are changing and reconceptualizing narratives regarding immigrants in the digital diaspora sphere is necessary.

The research manifested how in today’s digitalized world, narratives have taken on a different psychological and emotional dimension, leading to more severe forms of polarization, identity fragmentation, and emotional manipulation, as individuals and groups navigate competing stories in the struggle for meaning and belonging. Various studies reviewed in this research indicated the intersection of multiculturalism and social media functions as a double-edged sword for diasporic groups. While social media empowers racialized minorities and diaspora to amplify their voices and share their stories, it also provides a platform for anti-immigrant groups to further polarize society on issues such as nationalism, truth, and belonging. The research has identified three key findings regarding how social media influences the identity, narratives, and memory of diasporic communities:

(1) Within the dichotomy of positive potential and negative drawbacks, we could argue that social media is revealing its Janus-faced nature more than ever. While it facilitates connection and community-building, social media can also spread misinformation and create echo chambers that polarize communities. The reviewed literature indicates a shift from initial euphoria surrounding social media research to a sobering reality check. In this context, the vision of a digitally facilitated global village gives way to a dystopian view in which individuals and cultures are increasingly governed by algorithms and AI. This has implications for diasporic identity and memory on both personal and collective levels.
(2) Interactions with digital technology significantly influence personal identity and narrative formation. Digital platforms provide an opportunity for individuals to promote their narratives and tell their stories. However, a state of “disengaged engagement” (Han, 2024) arises from the constant flow of information, leading to commodified narratives and shallow interactions. The very idea of narrative itself is now viewed as being in crisis amid digitalization and information overload, as humans, once historical storytellers, have become social media “storysellers.”
(3) Digital technology and social media serve both to transition and preserve culture and memory with mixed implications. Profit-driven algorithms can homogenize experiences and reinforce dominant narratives, thereby undermining cultural diversity. Indeed, while digital platforms create a more connected world and foster cross-cultural interaction, they also raise concerns about cultural imperialism. Different communities mobilize around diverse identity markers like race, ethnicity, gender, language, or culture. Addressing how different groups leverage social media to negotiate their politics of identity requires a careful examination of the diverse ways in which these technologies impact cultural expressions and community dynamics. Furthermore, the transient nature of social media presents challenges for collective memory. Digital platforms risk losing important cultural archives due to their ephemeral content which can contribute to a form of “digital amnesia.”

In proposing a new analytical framework for studying the impact of digital narratives on collective memory and diasporic identities, it is essential to recognize that digital media has complicated our relationship with memory by simultaneously acting as a guardian and a threat to it; while we are encouraged to preserve our narratives, the overwhelming influx of information often leads to rapid forgetting and superficial recollection. This paradox can be investigated within diasporic communities, where identities are continuously reshaped through engagement with digital platforms that dissolve traditional boundaries of space and time. As these communities navigate their historical legacies and contemporary realities, their definitions become fluid, influenced by the dynamic interplay of digital narratives that both connect and fragment their collective experiences. Understanding this intricate relationship is crucial for comprehensively analyzing how digital storytelling not only reflects but also reconstructs the identities of individuals and communities in the diaspora.

## Limitations

Several limitations shape the scope of this analysis. First, the corpus is restricted to English-language academic publications, which likely excludes relevant scholarship in other languages and from underrepresented regions. Second, although efforts were made to include a diversity of diasporic groups and host societies, the sample is still skewed toward diasporas located in the Global North. Our intent was not to present a systematically diverse or equitably distributed sample of works representing various diasporic groups. In hindsight, however, we recognize that adopting such an approach from the outset could have strengthened the structure and inclusivity of our analysis. Third, the academic nature of the texts included may underrepresent community-based knowledge or alternative storytelling practices that fall outside of formal research contexts. These limitations do not diminish the value of the corpus but do suggest directions for future research, which we discuss in more detail below.

## Research recommendations

In examining the relationship between social media narratives, diasporic identity, and collective memory, three key areas for future research can be proposed. First, a comparative analysis of digital narratives across various diasporic communities could offer critical insights into how different groups use digital platforms to construct and transmit their collective memories. Such an analysis would not only identify distinct storytelling strategies, aesthetic choices, and cultural references but also illuminate how different historical experiences, migration trajectories, and degrees of marginalization shape the ways diasporic groups remember and represent the past. It would also allow for an understanding of intergenerational differences in narrative forms and media preferences.

Second, further research on the role of algorithms and platform dynamics in memory formation is essential. Social media algorithms determine the visibility, reach, and longevity of digital content, thereby influencing which diasporic narratives are amplified and which are marginalized or erased. Studying how algorithmic curation affects the preservation, repetition, and forgetting of collective memories can deepen our understanding of the politics of memory in digital spaces. This includes examining how diasporic users strategically navigate platform logics to make their histories visible or engage in memory work that resists dominant media representations.

Third, investigating the intersection of digital activism and collective memory can reveal how diasporic communities mobilize around shared histories to advocate for justice, recognition, or political change. Digital activism often involves reclaiming suppressed or silenced narratives, commemorating traumatic events, and countering official histories. Research in this area could explore how diasporic activists use hashtags, digital art, oral history archives, or online campaigns to create affective memory publics that challenge hegemonic discourses and foster solidarity across borders. Together, these areas of inquiry could significantly advance our understanding of how digital narratives mediate identity, belonging, and memory within and across diasporic communities in the contemporary media landscape.

## Data Availability

Data sharing is not applicable as no new data were generated or analysed during this study*.

## Note

1 We use the term *host society* with caution. Although it is widely used in migration and diaspora studies, it carries normative implications that may reinforce a guest–host binary, implicitly positioning diasporic individuals as outsiders—even in countries where they are full citizens. We use the term here for consistency with the existing literature, while remaining critically aware of its limitations.
